# Remote multimodality monitoring of maternal physiology from the first trimester to postpartum period: study results

**DOI:** 10.1097/HJH.0000000000003260

**Published:** 2022-08-12

**Authors:** Agata P. Zielinska, Edward Mullins, Elena Magni, Giulia Zamagni, Hana Kleprlikova, Olive Adams, Tamara Stampalija, Lorenzo Monasta, Christoph Lees

**Affiliations:** a Department of Metabolism, Digestion and Reproduction, Imperial College London; b Queen Charlotte's and Chelsea Hospital, Imperial College Healthcare NHS Trust; c The George Institute for Global Health, London, UK; d Institute for Maternal and Child Health – IRCCS “Burlo Garofolo”, Trieste, Italy; e Department of General Anthropology, Faculty of Humanities, Charles University in Prague, Czech Republic; f Department of Medical, Surgical and Health Sciences, University of Trieste, Trieste, Italy

**Keywords:** blood pressure, heart rate, home monitoring, pregnancy, prenatal care, weight

## Abstract

Current antenatal care largely relies on widely spaced appointments, hence only a fraction of the pregnancy period is subject to monitoring. Continuous monitoring of physiological parameters could represent a paradigm shift in obstetric care. Here, we analyse the data from daily home monitoring in pregnancy and consider the implications of this approach for tracking pregnancy health.

**Methods:**

Prospective feasibility study of continuous home monitoring of blood pressure, weight, heart rate, sleep and activity patterns from the first trimester to 6 weeks postpartum.

**Results:**

Fourteen out of 24 women completed the study (58%). Compared to early pregnancy [week 13, median heart rate (HR) 72/min, interquartile range (IQR) 12.8], heart rate increased by week 35 (HR 78/min, IQR 16.6; *P* = 0.041) and fell postpartum (HR 66/min, IQR 11.5, *P* = 0.021). Both systolic and diastolic blood pressure were lower at mid-gestation (week 20: SBP 103 mmHg, IQR 6.6; DPB 63 mmHg, IQR 5.3 *P* = 0.005 and *P* = 0.045, respectively) compared to early pregnancy (week 13, SBP 107 mmHg, IQR 12.4; DPB 67 mmHg, IQR 7.1). Weight increased during pregnancy between each time period analyzed, starting from week 15. Smartwatch recordings indicated that activity increased in the prepartum period, while deep sleep declined as pregnancy progressed.

**Conclusion:**

Home monitoring tracks individual physiological responses to pregnancy in high resolution that routine clinic visits cannot. Changes in the study protocol suggested by the study participants may improve compliance for future studies, which was particularly low in the postpartum period. Future work will investigate whether distinct adaptative patterns predate obstetric complications, or can predict long-term maternal cardiovascular health.

## INTRODUCTION

In pregnancy, maternal physiology undergoes a set of adaptations that begin shortly after conception [1–3]. Sub-optimal adaptation to pregnancy may not only compromise fetal development and maternal health, but is also associated with worse long-term health outcomes [1,4–6]. Currently, obstetric care largely detects such abnormalities by relying on the woman reporting concerning symptoms to the clinician or the recognition of worrying signs during scheduled antenatal appointments [7], which are often widely spaced. Worldwide, 41.4% of women do not attend an antenatal appointment by the end of the first trimester [8] and even in the UK, scheduled antenatal care predicts that a pregnant woman interacts with healthcare professionals only nine to 11 times during the whole pregnancy [9].

The infrequent nature of antenatal appointments may limit the ability to detect early signs of pregnancy compromise. Digital health is the use of information and communication technologies to manage health risks and promote wellbeing [10]. Remote monitoring using digital devices has been previously successfully employed to manage chronic conditions such as cardiovascular disease and diabetes [11,12], and remote monitoring of pulse oximetry readings in the community has been recently utilized to identify early signs of deterioration in patients with coronavirus disease 2019 (COVID-19) [13]. However, digital health is not commonly used in routine pregnancy care. Pregnant women could be excellent candidates for remote health monitoring, as they are generally thought to be more motivated to implement lifestyle changes [14], and some serious pregnancy complications are preceded by early warning signs [3,15]. Thus, continuous monitoring of basic physiological parameters could offer a paradigm shift in the field of pregnancy care.

In particular, the cardiovascular system offers a unique window into monitoring the wellbeing of the mother and the fetus. As this system adapts at different stages of gestation to fulfill the changing nutrient and oxygen demands of the feto-maternal unit, cardiovascular parameters adapt in a stepwise manner [1]. In particular, the remodeling of maternal spiral arteries at the end of the first trimester increases the blood flow through the placenta [15–17]. Failure to remodel spiral arteries is associated with pregnancy conditions, such as preeclampsia, miscarriage or intra-uterine growth restriction [4,18–25], though more recently it has been shown that cardiovascular function prior to pregnancy may also contribute to these conditions [20]. As cardiovascular parameters such as heart rate (HR), systolic blood pressure (SBP), diastolic blood pressure (DBP) and stroke volume can be monitored in a noninvasive manner, pathological adaptations of this system might be readily detected, offering the potential for lifestyle and/or pharmacological intervention.

Body weight changes markedly during pregnancy, is closely associated with cardiovascular function and can be monitored noninvasively. During pregnancy, body weight ought to increase as the fetus grows and structures that support pregnancy develop [1]. However, a proportion of women deposit a disproportionate amount of visceral fat while pregnant [26]. The Institute of Medicine (IOM) recommends a weight gain of 11.5–16.0 kg during a typical pregnancy [27]. Over 50% of pregnancies appear to exceed the recommended gestational weight gain (GWG) [28] and an excessive GWG leads to short and long-term consequences for population health. For the mother, pronounced GWG may result in pregnancy-associated hypertension, gestational diabetes and a complicated delivery [27,28]. Moreover, it can have long-term consequences for a women's health, as it is an important predictor of obesity in later life [29]. From the baby's perspective, excessive GWG not only worsens health at birth, but may also lead to obesity that projects into adulthood [28,30–32]. Thus, to maximize health across generations, excessive GWG should be recognized early in pregnancy and addressed before fetal development is completed. Except at pregnancy booking, weight is not routinely measured in pregnant women in the UK. Hence, it is axiomatic that excess weight gain cannot be identified.

Our experience showed that monitoring hemodynamic parameters such as SBP, DBP, HR, and activity levels from the comfort of one's home is feasible. In the present article, we analyze the generated trajectories from first trimester to the postpartum period, consider the implications of these for an individual mother's health and how these results could inform the design of future population-based studies of continuous monitoring of health in pregnancy.

## METHODS

### Ethical considerations

The study obtained ethical approval from the London Fulham Regional Ethics Committee and Health Research Authority (HRA; IRAS ID 233138). Each participant gave a written consent and was deemed to have capacity to make decisions regarding study participation.

### Design

Prospective feasibility study of remote daily monitoring of SBP, DBP, HR, activity level, sleep pattern and body weight from first trimester to 6 weeks postpartum. This manuscript evaluates the measurements obtained during the course of this feasibility study.

### Participant recruitment

**FIGURE 1:**
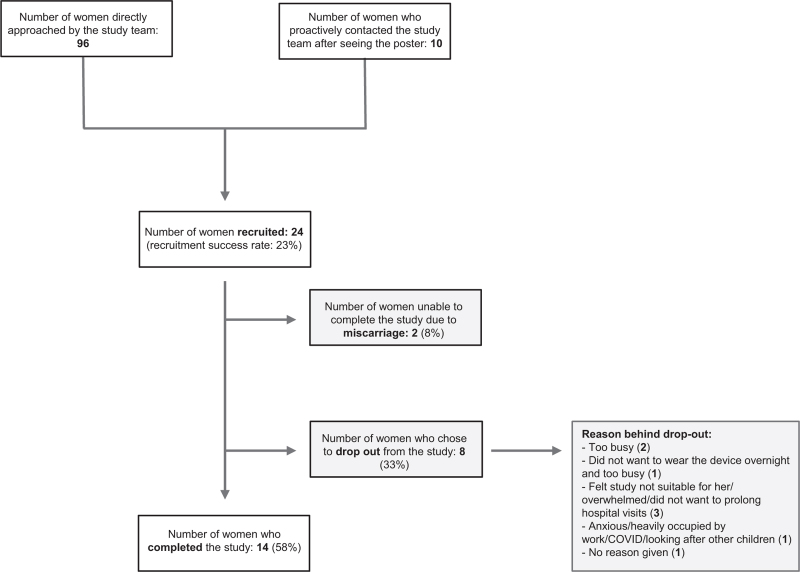
Flow chart demonstrating recruitment strategy, success rates and the main reasons behind participant drop-out.

Posters advertising the study were placed in the hospital's maternity unit and a nearby community antenatal clinic, including common areas (bathrooms, lifts) and the antenatal ward. Potential participants were invited to phone or E-mail a member of the study team to express an interest in participating in the study. Additionally, study team members approached potential participants directly in the waiting room of the hospital's antenatal clinic and a community antenatal clinic. In total, of 10 women who saw the poster and 96 women directly approached by the study team members, 24 women were ultimately recruited for the study (Fig. 1).

### Protocol for remote daily monitoring of cardiovascular parameters in pregnant women

Individuals recruited for the study consented to daily home monitoring of bodily physiology, attending four clinical appointments and completing study questionnaires at three predetermined intervals. To be recruited to the study, individuals had to fulfill the following criteria: age 18–45 years, ≤12 weeks pregnant at the time of recruitment, provision of an informed consent and communicative English. All participants were provided with a blood pressure machine (Microlife, BP A1 EASY), weight scale (Tristar WG-2421) and a Fitbit Inspire HR fitness tracker watch for daily self-monitoring. The smartwatch recorded the following parameters: HR, activity, steps taken, time awake, time asleep and light/deep/REM sleep duration. Activity was automatically detected and categorized by the smartwatch if the participant was burning three times as many calories as at rest (Smarttrack, Fitbit's proprietary algorithm) [33]. The Fitbit device has been previously validated in other patient groups, including for step count measurements [34]. As per the study protocol, the women were asked to continuously wear the smartwatch throughout pregnancy and the postpartum period, and record once daily SBP, DBP and body weight. A mobile app (Huma [35], London, UK – formerly Medopad) was provided to each study participant to record the measurements. SBP, DBP and body weight measurements were manually entered into the mobile phone app, whereas smartwatch recordings (HR, sleep patterns and activity) were automatically transferred via Bluetooth.

### Addressing abnormal recordings

Participants were given written information about what values indicate an abnormal blood pressure reading (SBP > 140 mmHg, DBP > 90 mmHg on at least two separate occasions), so that they could seek medical advice. The recordings entered into the app were monitored remotely by the study team, so that readings of potential significance could be identified. The blood pressure measurement was taken as an average of three readings in clinic, and a single reading at home.

### Study outcomes

The primary outcome of this study was to determine the feasibility of employing home monitoring devices to track health in pregnancy and to gain a comprehensive profile of changes in physiological parameters from first trimester to the postpartum period.

### Sample size

This manuscript describes the results of a feasibility study, and hence a formal power calculation was not performed as part of the study design.

### Generation of pregnancy plots for individual patients

Pregnancy plots were generated in Origin Pro (Origin Labs) based on values recorded by study participants and stored in Excel (Microsoft). All values available were included in the analysis.

### Generation of percentile curves

The percentile curve estimation was performed using GAMLSS package (R) with Box-Cox Cole & Green (BCCG), Box-Cox Power Exponential (BCPE) and Box-Cox *t* (BCT) distributions for the variables of interest (HR, SBP, DBP, body weight). To construct the HR, SBP and DBP curves, data from all patients were included. To construct the curves for body weight, all but one participant who was an outlier (BMI 40 and only 17 body weight measurements tabulated, in contrast to an average of 148 measurements for other individuals in the study cohort) were included. To study the differences in parameters across gestations, individuals with missing data were excluded from the final analysis (*N* = 3/14 for HR, SBP and DBP, and *N* = 5/14 for body weight) as for the Wilcoxon paired signed-rank test, observations at both timepoints of interest for each study individual has to be available for analysis. Thus, only patients with a minimum of one measurement per week for weeks: 13, 15, 20, 25, 30, 35 and postpartum were included in the final statistical analysis. Cubic splines and penalized splines with different degrees of freedom were added to model the scale and shape parameters. For each variable of interest, multiple models were estimated adopting different splines for every candidate distribution (BCCG, BCPE, BCT). The Akaike's information criterion (AIC) was used to select the best model (i.e. the one with the lowest deviance).

For sleep and activity patterns across pregnancy, weekly averages were calculated for each parameter of interest. Because of the high variability in the data across study participants, the trend over the gestation period was then modeled using a nonparametric approach. In particular, smoothed conditional means were estimated using local weighted regression (LOESS).

### Statistical analysis of change in parameters (heart rate, systolic blood pressure, diastolic blood pressure, body weight) at different pregnancy timepoints

To evaluate the accuracy of home monitoring devices, Bland-Altman plots were generated for all datapoints where a home measurement and a clinic measurement occurred within a 48-h period. To compare how physiological parameters change across pregnancy, weekly data were obtained for each patient by computing the average of the daily observations. Postpartum data were obtained for each patient by taking the mean value of all postpartum observations. To study the changes in cardiovascular parameters during pregnancy, initially 5-week intervals from week 10 to week 40 were considered. As only seven of 14 patients had some data for week 10 and only five of 40 for week 40 and the Wilcoxon signed-rank test relies on paired comparisons at different time points, week 10 and 40 were excluded from the analysis to re-dimension the dataset and gain a large number of patients with nonmissing data available for analysis (*N* = 11/14). Wilcoxon signed-rank test's *P*-values: ^∗∗∗^*P* < 0.001, ^∗∗^*P* < 0.01, ^∗^*P* < 0.05.

## RESULTS

### Adherence to the study protocol

Twenty-four women were recruited to the study (median age 32, interquartile range [IQR] = 28.5–37.5), 50% of whom were Caucasian. Fourteen out of 24 women completed the study (58%). Two women were unable to complete the study due to a miscarriage (8%), and eight did not complete (33%), mainly because the women were too busy or feeling overwhelmed (Figure 1). On average, each study participant who completed the study took 4.3 home recordings of each modality per week (standard deviation [SD] = 2.20) during the pregnancy period and 2.0 recordings per week during the 6-week postpartum period (SD = 2.41, recommended number of recordings per week as per the study protocol = 7). Six out of 14 participants when asked about the missing data highlighted the difficulty in obtaining regular recordings during the postdelivery period (Table 1). Additionally, study participants emphasized that manual data recording for body weight and blood pressure was troublesome (Table 1).

**TABLE 1:** Summary of the study's exit questionnaire, which highlights study caveats and the reasons behind imperfect adherence to the study protocol

Study participants	Question A: If you were not able to record some of the information, what was the reason?
1	Fitbit watch. Being too tired/busy
2	Difficult after baby born/no time
3	Not recording when away from home. More difficult to record after birth, as little time
4	Too tired/forgot. Difficult to do it after baby was born and often in my arms
5	No time after baby is born, as it took 10 min each time to enter the data
6	Fatigue/child care
7	Falling asleep before entering the results
8	Too busy/noisy BP device
9	Fitbit battery needing charging. When in hospital - forgetting to input data
10	Forgot/other priorities
11	Forgot/sleeping
12	Various reasons. Post pregnancy I was getting little sleep, so I did not want the watch to wake me
13	When travelling. Needed to build it into daily routine at home. Postdelivery: much more difficult as no routine
14	Fitbit issues - recording and syncing

	Question B: How often would you suggest to ask women to check these parameters, not to interfere with their everyday life?
1	Weekly
2	Time was an issue
3	For BP, pulse and weight - could be twice daily
4	Once a week for BP, weight pulse. Daily for activity, steps and sleep
5	Weight is hard to track as you gain extra weight in pregnancy, but maybe once per week
6	Once a day, ask women to do it first thing in the morning
7	BP daily, weight - less often (weekly/every two weeks). Pulse/sleep/steps – daily
8	Every day
9	Steps daily, other parameters - weekly/twice a week
10	Three times a week
11	Daily (would be even harder to remember if other days)
12	Every other day
13	BP and weight twice a week. Fitbit daily plus download the data directly to the Medopad (not to have to enter it manually)
14	Weekly

### Home monitoring recordings reliably reflect clinic readings

**FIGURE 2:**
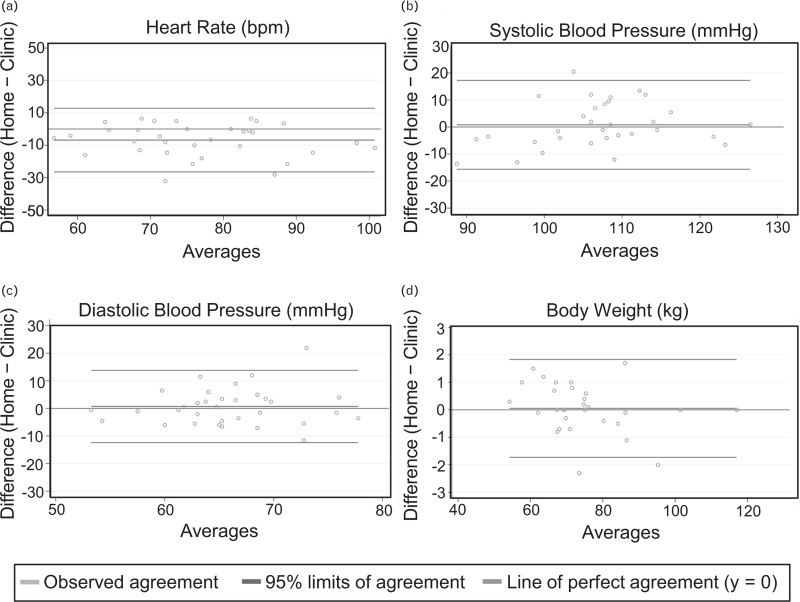
Correlation between home measurements and clinic measurements. Bland–Altman plots for: (a) HR (beats/min); (b) SBP (mmHg); (c) DBP (mmHg); (d) body weight (kg). Only cases where recordings for both home and clinic measurements recorded within a 48-h period were available are included in the analysis. Green – line of perfect agreement, *y* = 0; purple – observed agreement, red – 95% limits of agreement.

We first investigated how home recordings were related to measurements taken by medical professionals in a clinical setting. For this purpose, we compared the values for body weight, SBP and DBP for cases where both clinic and home measurements were taken within a 48 h period (Fig. 2a–d). This revealed that the measurements obtained at home were not significantly different from those surveyed in the clinic: weight was on average higher for the home recordings by 0.05 kg (SD = 0.91, 95% limits of agreement: −1.73–1.83), SBP by 0.83mmHg (SD = 8.39, 95% limits of agreement: −15.61–17.26) and DBP by 0.70 mmHg (SD = 6.68, 95% limits of agreement: −12.39–13.78). Of note, during the study duration only 4/1956 (0.2%) home systolic blood pressure recordings that were entered to the app exceeded 140 mmHg SBP.

### Frequent monitoring reveals how an individual adapts to different stages of pregnancy

Patient-specific curves for individuals’ HR, SBP and DBP changes are shown in Figure 1, Supplemental Digital Content.

No patients consistently had blood pressure recordings higher than 140/90 mmHg between 20 weeks’ gestation and the onset of labor; this was in line with the clinical information extracted from patients’ medical records, which showed that none developed gestational hypertension or preeclampsia (Table 2).

**TABLE 2:** Frequency of pregnancy complications and neonatal outcomes across the study cohort

Characteristic	Proportion of the cohort
Gestational hypertension	0/14 (0%)
Preeclampsia	0/14 (0%)
Gestational diabetes	3/14 (21.40%)
Fetal size on 20w US	Normal: 14/14 (100%)
Gestation at birth	39 + 4 (38 + 3 – 41 + 1, *n* = 14)
Birthweight (g)	3457 g (SD = 524, *n* = 14)
Neonatal birth outcome	No maternal concerns: 14/14 (100%) Apgar score at 1 min: 9 (9–9, *n* = 9) Apgar score at 5 min: 10 (9–9, *n* = 9)

Data in the table is presented as median (IQR). SD, standard deviation.

### Trends in heart rate across the patient cohort revealed by high-resolution cardiovascular trajectories

**FIGURE 3:**
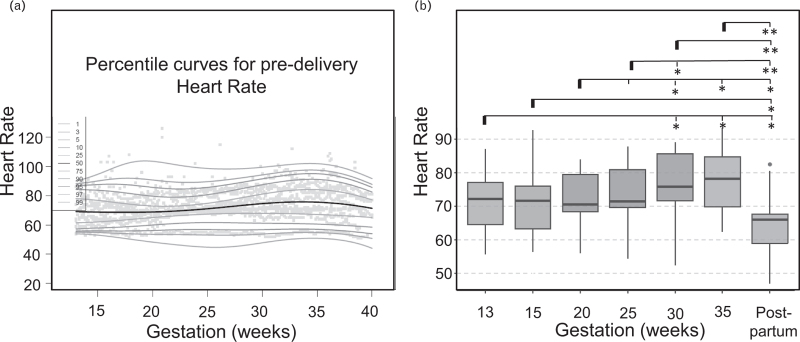
Trends in HR captured by home recordings. (a) HR percentile curves across gestations for the entire patient cohort; (b) Box plots summarizing how heart rate changes across gestations in the study cohort. For each line, the significance level is measured as compared to the first gestation highlighted (marked with a bold line). HR, heart rate.

We used the high-resolution data across the patient cohort (Figure 1, Supplemental Digital Content) to investigate conserved trends in cardiovascular changes during pregnancy (Fig. 3a). Analysis of HR trajectories revealed that across the patient cohort, HR significantly increased from early pregnancy (week 13, median 72.2/min, IQR = 12.8) to third trimester (week 30, median 75.8/min, IQR = 16.0; *P* = 0.033; Fig. 3b and Table 3). HR also significantly increased from week 20 (median = 70.5/min, IQR = 13.9) to week 30 (median = 75.8/min, IQR = 16.0; *P* = 0.023) and week 35 (median = 78.3/min, IQR = 16.6; *P* = 0.021; Fig. 3b), decreased from week 35 to the postpartum period (median = 66.0/min, IQR = 11.5; *P* = 0.003, Fig. 3B), and HR postpartum was significantly lower than during any gestational timepoint analyzed (Fig. 3B).

**TABLE 3:** Summary table of individual participants’ heart rate values captured by home monitoring across specific weeks of gestation

Heart rate (HR, bpm)	Week 13	Week 15	Week 20	Week 25	Week 30	Week 35	Postpartum
Participant 1	77.00 (1.0)	76.5 (1.9)	82.4 (3.3)	82.0 (3.7)	89.0 (9.5)	93.5 (5.0)	66.8 (5.5)
Participant 2	84.3 (4.6)	84.3 (8.7)	81.6 (0.6)	84.3 (0.8)	87.0 (0.8)	85.3 (1.0)	68.0 (4.2)
Participant 3	75.3 (8.1)	-	74.0 (10.4)	71.0 (1.2)	69.8 (3.1)	70.5 (1.7)	95.0 (4.2)
Participant 4	74.0 (9.1)	71.7 (1.1)	70.0 (2.9)	71.5 (2.0)	75.8 (2.6)	68.7 (2.1)	56.5 (3.1)
Participant 5	65.7 (2.1)	67.3 (1.0)	70.5 (6.2)	67.7 (0.5)	64.7 (1.0)	63.9 (0.9)	61.5 (2.1)
Participant 6	–	86.0 (0)	–	–	–	–	72.0 (2.8)
Participant 7	55.7 (1.9)	56.4 (1.6)	56.1 (2.6)	54.6 (0.5)	52.6 (1.0)	62.6 (14.4)	47.1 (4.4)
Participant 8	77.1 (2.7)	75.6 (2.3)	77.2 (3.7)	80.0 (1.7)	84.4 (0.9)	84.3 (1.4)	82.5 (2.1)
Participant 9	87.0 (1.4)	92.7 (10.5)	84.0 (0)	87.7 (4.6)	88.7 (3.1)	89.5 (0.7)	80.5 (9.2)
Participant 10	–	–	123.0 (4.2)	–	92.0 (0)	98.0 (0)	99.0 (7.0)
Participant 11	64.3 (2.5)	62.6 (0.8)	67.7 (1.5)	69.7 (1.0)	82.0 (0)	82.0 (0)	67.3 (4.2)
Participant 12	64.8 (0.8)	64.1 (0.4)	63.7 (1.6)	69.5 (0.6)	72.2 (0.8)	72.2 (0.5)	62.7 (4.7)
Participant 13	59.9 (1.6)	59.7 (0.8)	71.3 (9.6)	72.9 (0.7)	75.6 (1.0)	78.3 (1.1)	54.5 (8.9)
Participant 14	72.2 (1.6)	73.3 (1.5)	69.0 (0)	71.0 (0)	71.0 (0)	71.0 (0)	66.0 (0)
Across the participant cohort	Median 72.2 (IQR = 64.3–77.1)	71.7 (62.6–76.5)	70.5 (67.7–81.6)	71.5 (69.5–82.0)	75.8 (71.0–87.0)	78.3 (68.7–85.3)	66.0 (56.5–68.0)

^∗^Values for individual participants are reported as mean (SD). For Wilcoxon signed-rank test, individuals with missing data for the weeks analyzed (participants 3, 6 and 10) had to be excluded from the statistical analysis.

### Trends in blood pressure across the patient cohort revealed by high-resolution clinical observation trajectories

**FIGURE 4:**
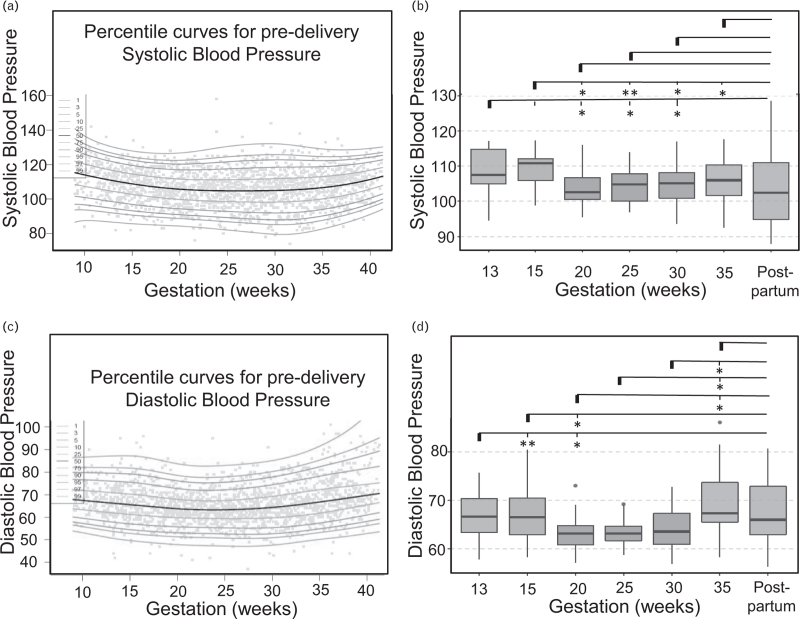
Trends in SBP and DBP captured by home recordings. (a) SBP percentile curves across gestations for the entire patient cohort; (b) Box plots summarizing how SBP changes across gestations in the study cohort; (c) DBP percentile curves across gestations for the entire patient cohort; (d) Box plots summarizing how DBP changes across gestation in the study cohort. For each line, the significance level is measured as compared to the first gestation highlighted (marked with a bold line).

Analysis of SBP trends (Figure 1, Supplemental Digital Content) across the patient cohort (Fig. 4a) revealed that there was a significant drop in blood pressure from early pregnancy (week 13, median SBP = 107.4 mmHg, IQR = 12.4) to mid-gestation (week 20, SBP = 102.7 mmHg, IQR = 6.6; *P* = 0.045; Fig. 4b and Table 4). SBP remained significantly lower also across later gestations (week 25, SBP = 104.8 mmHg, IQR = 9.5 and week 30, SBP = 105.2 mmHg, IQR = 12.0; *P* = 0.016 for both when compared to week 13, Fig. 4b), only to return to early pregnancy values by week 35 (SBP = 106.0 mmHg, IQR = 13.0; *P* = 0.075, NS; Fig. 4b). SBP postpartum (SBP = 102.5 mmHg, IQR = 22.6) was not significantly different from the pregnancy period.

**TABLE 4:** Summary table of individual participants’ systolic blood pressure values captured by home monitoring across specific weeks of gestation

Systolic blood pressure (SBP, mmHg)	Week 13	Week 15	Week 20	Week 25	Week 30	Week 35	Postpartum
Participant 1	115.7 (0.6)	110.8 (1.7)	102.7 (8.1)	109.5 (6.2)	109.0 (0)	111.0 (9.9)	106.4 (14.1)
Participant 2	100.1 (5.4)	100.1 (5.6)	100.4 (4.8)	100.1 (5.5)	105.2 (6.8)	96.3 (4.6)	102.6 (7.1)
Participant 3	106.8 (9.4)	–	107.0 (6.9)	98.8 (10.3)	104.8 (17.9)	116.3 (11.3)	111.5 (3.5)
Participant 4	103.3 (4.3)	103.6 (4.5)	95.8 (7.2)	100.0 (10.9)	93.7 (7.0)	92.5 (4.4)	96.7 (4.5)
Participant 5	107.0 (1.4)	112.0 (7.9)	107.0 (5.6)	97.0 (1.4)	97.0 (15.6)	106.0 (6.7)	128.4 (6.8)
Participant 6	–	134.0 (0)	–	–	–	–	97.5 (4.9)
Participant 7	115.9 (2.3)	111.1 (4.6)	106.6 (5.9)	111.0 (4.9)	111.4 (4.2)	105.9 (4.1)	119.8 (8.5)
Participant 8	107.4 (7.0)	110.8 (7.7)	102.3 (3.9)	104.0 (7.0)	104.8 (8.9)	112.2 (7.8)	102.5 (0.7)
Participant 9	113.0 (2.8)	112.3 (3.1)	116.0 (0)	105.3 (3.8)	107.3 (5.5)	108.0 (2.8)	93.0 (2.8)
Participant10	–	–	110.5 (17.7)	–	124.0 (0)	128.0 (0)	110.7 (1.5)
Participant 11	117.1 (5.3)	117.0 (2.8)	114.6 (6.2)	113.9 (5.0)	117.0 (5.3)	117.5 (3.9)	115.6 (5.5)
Participant 12	94.6 (8.8)	98.8 (6.6)	95.5 (5.4)	98.0 (7.8)	94.0 (8.5)	98.0 (3.7)	88.0 (8.4)
Participant 13	113.9 (2.3)	117.3 (4.9)	102.8 (6.9)	106.1 (6.2)	104.9 (4.7)	109.7 (4.3)	102.1 (6.0)
Participant 14	106.7 (3.9)	108.3 (3.3)	100.6 (3.0)	104.8 (3.3)	105.7 (4.0)	105.3 (5.3)	92.0 (2.8)
Across the participant cohort	Median 107.4 (IQR = 103.3–115.7)	110.8 (103.6–112.3)	102.7 (100.4–107.0)	104.8 (100.0–109.5.0)	105.2 (97.0–109.0)	106 (98.0–111.0)	102.5 (93.0–115.6)

^∗^Values for individual participants are reported as mean (SD). For Wilcoxon signed-rank test, individuals with missing data for the weeks analyzed (participants 3, 6 and 10) had to be excluded from the statistical analysis.

Analysis of DBP trends (Fig. 4c) also revealed a significant drop in blood pressure when early pregnancy (week 13; median DBP = 66.7 mmHg, IQR = 7.1) was compared to mid-gestation (week 20, DBP = 63.2 mmHg, IQR = 5.3; *P* = 0.005, Fig. 4d and Table 5). DBP remained significantly lower by week 25 (DBP = 63.2 mmHg, IQR = 3.8; *P* = 0.041, Fig. 4d), but returned to early pregnancy values earlier than SBP (week 30 DBP = 63.6 mmHg, IQR = 9.2, *P* = 0.091; NS, Fig. 4d). Moreover, DBP increased at around week 35 (DBP = 67.3, IQR = 10.2), which was significant with respect to all weeks 20, 25 and 30 (*P* = 0.016, *P* = 0.013 and *P* = 0.041, respectively; Fig. 4d). As for SBP, DBP in the postpartum period (DBP = 66.0, IQR = 14.8) was not significantly different from the pregnancy period.

**TABLE 5:** Summary table of individual participants’ diastolic blood pressure values captured by home monitoring across specific weeks of gestation

Diastolic blood pressure (DBP, mmHg)	Week 13	Week 15	Week 20	Week 25	Week 30	Week 35	Postpartum
Participant 1	66.7 (2.1)	63.3 (1.5)	62.7 (1.5)	64.5 (2.9)	62.0 (0)	86.0 (15.6)	69.6 (7.3)
Participant 2	63.4 (2.1)	66.6 (5.5)	63.2 (4.1)	62.3 (3.5)	65.6 (5.0)	59.4 (2.6)	69.7 (3.7)
Participant 3	63.0 (14.2)	–	62.0 (4.6)	64.5 (11.7)	62.8 (7.8)	75.3 (11.5)	75.5 (0.7)
Participant 4	60.3 (1.7)	68.0 (12.1)	59.0 (11.9)	58.9 (9.8)	59.8 (4.1)	58.3 (3.1)	61.2 (4.5)
Participant 5	70.5 (3.5)	70.0 (7.1)	65.3 (5.0)	63.0 (7.1)	57.0 (12.7)	66.0 (5.7)	76.4 (3.8)
Participant 6	–	83.0 (0)	–	–	–	–	56.5 (2.1)
Participant 7	69.6 (2.4)	62.6 (7.6)	61.8 (4.3)	64.1 (1.6)	64.9 (2.3)	68.8 (5.1)	80.5 (5.3)
Participant 8	75.6 (5.9)	79.0 (3.2)	69.0 (2.2)	69.2 (4.2)	71.5 (2.5)	81.4 (4.6)	76.0 (0)
Participant 9	73.0 (0)	80.3 (11.2)	73.0 (0)	69.0 (1.7)	72.7 (2.5)	75.5 (0.7)	66.0 (4.2)
Participant 10	–	–	72.5 (6.4)	–	78.0 (0)	79.0 (0)	73.7 (4.9)
Participant 11	63.4 (3.6)	62.0 (3.8)	63.4 (7.3)	64.9 (4.4)	69.0 (1.7)	67.3 (3.2)	65.8 (4.8)
Participant 12	57.9 (5.9)	58.4 (5.5)	57.3 (4.1)	60.6 (8.4)	57.8 (7.5)	71.8 (13.0)	56.4 (6.6)
Participant 13	70.3 (2.0)	70.9 (4.1)	64.3 (5.2)	61.1 (2.4)	62.6 (3.4)	65.7 (4.3)	64.7 (3.6)
Participant 14	64.5 (10.7)	64.8 (3.1)	60.0 (2.5)	63.2 (1.6)	63.6 (1.9)	65.3 (6.1)	56.5 (0.7)
Across the participant cohort	Median 66.7 (IQR = 63.4–70.5)	66.6 (62.6–70.9)	63.2 (60.0–65.3)	63.2 (61.1–64.9)	63.6 (59.8–69.0)	67.3 (65.3.0–75.5)	66.0 (61.2–76)

^∗^Values for individual participants are reported as mean (SD). For Wilcoxon signed-rank test, individuals with missing data for the weeks analyzed (participants 3, 6 and 10) had to be excluded from the statistical analysis.

### Trends in body weight across gestation

Plotted weight trajectories for individual patients across the trimesters are shown in Figure 2, Supplemental Digital Content. For 13/14 patients, we had sufficient data to calculate total pregnancy weight gain (GWG). GWG ranged between 5.6 and 42 kg across the cohort (Table 6; mean = 16.64 kg, SD = 9.68 kg).

**TABLE 6:** Table summarizing weight gain in pregnancy for the study cohort, stratified by prepregnancy BMI

Baseline BMI	Total weight gain (kg)		Absolute gain in 1st trimester (kg)		Rate of gain (kg/week) in 2^nd^ and 3^rd^ trimesters		
	Guideline	Observed	Guideline	Observed	Guideline	Observed2^nd^	Observed 3^rd^
Normal (6)	11.5–16	15.4 (11.9–18.0)	0.5–2	3.8 (2.3–7.5)	0.35–0.50	0.4 (0.4–0.5)	0.2 (0.2–0.3)
Overweight (5)	7–11.5	13.5 (12.8–30.2)	0.5–2	1 (0.7–5.5)	0.23–0.33	0.4 (0.3–0.6)	0.7 (-0.01–0.8)
Obese (2)	5–9	10.2 (7.9–12.5)	0.5–2	0.4 (-0.4–0.8)	0.17–0.27	0.4 (0.3–0.5)	0.4 (0.2–0.4)

In the “Observed” columns, data is presented as median (Q1–Q3).

Analysis revealed that 63.6% of the study cohort exceeded recommended IoM weight gain [27] within the first trimester (Table 6, middle column), while 45.6% showed an increased gain weight rate in second and third trimesters. For individuals with normal prepregnancy BMI, the rate of weight gain was higher in the second than the third trimester, while the trend was reversed for women with BMI in the overweight category (Table 6, right panel).

**FIGURE 5:**
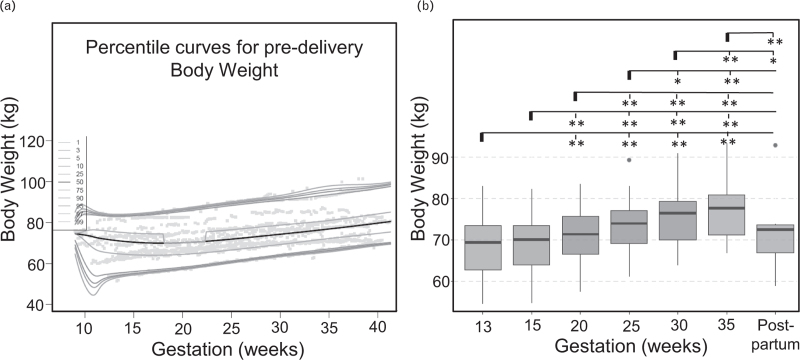
Trends in body weight captured by home recordings. (a) Body weight percentile curves across gestations in the study cohort; (b) Box plots summarizing how body weight changes across gestations across the patient cohort. For each line, the significance level is measured as compared to the first gestation highlighted (marked with a bold line).

Analysis of the cumulative curves generated from values recorded across the patient cohort (Fig. 5a) revealed that weight increased significantly during pregnancy between each time period analyzed, starting from week 15 (Fig. 5b and Table 7).

**TABLE 7:** Summary table of individual participants’ body weight values captured by home monitoring across specific weeks of gestation

Weight (kg)	Week 13	Week 15	Week 20	Week 25	Week 30	Week 35	Postpartum
Participant 1^∗^	69.4 (0.1)	70.1 (0.3)	71.4 (0.4)	73.9 (0.7)	76.6 (0.5)	80.8 (0.4)	73.2 (1.7)
Participant 2	73.3 (0.5)	72.3 (1.1)	75.7 (0.3)	77.0 (0.4)	79.4 (0.3)	80.9 (0.6)	73.8 (1.2)
Participant 3	81.6 (0.5)	–	86.5 (0.6)	90.4 (0.2)	–	–	90.6 (0)
Participant 4	65.1 (0.2)	65.7 (0.5)	67.4 (0.2)	69.4 (0.8)	70.2 (0.3)	70.8 (0.2)	68.2 (1.3)
Participant 5^∗^	73.5 (0.3)	73.4 (0.1)	75.1 (0.1)	77.0 (0.2)	76.5 (0.3)	77.7 (0.3)	72.5 (0.9)
Participant 6	–	74.7 (0)	–	–	–	–	69.7 (0)
Participant 7	59.0 (5.0)	63.5 (0.5)	66.5 (0.5)	69.1 (0.5)	70.0 (0.4)	71.2 (0.4)	64.5 (0.8)
Participant 8	68.3 (1.1)	–	67.8 (0)	71.7 (0.4)	74.7 (1.1)	77.6 (0.2)	76.2 (0)
Participant 9^∗^	77.0 (0.4)	77.2 (0.7)	79.2 (0)	83.0 (0.4)	85.3 (0.3)	84.8 (0.2)	73.7 (0)
Participant 10	–	–	119.5 (0)	–	123.2 (0)	122.0 (0)	121.0 (0)
Participant 11	83.0 (0)	82.3 (0.2)	83.6 (1.7)	89.4 (0.2)	90.9 (0.1)	93.4 (0.1)	92.9 (1.3)
Participant 12	63.9 (0.7)	65.5 (0.5)	69.2 (0)	72.4 (0.1)	72.7 (0)	–	66.6 (1.1)
Participant 13	54.5 (0.2)	54.8 (0.3)	57.6 (0.4)	61.2 (0.3)	64.0 (0.5)	66.8 (0.3)	58.8 (1.6)
Participant 14	62.7 (0.5)	63.9 (0.4)	65.5 (0.4)	67.1 (0.4)	69.6 (0.5)	72.3 (0.2)	66.9 (1.0)
Across the participant cohort	Median 69.4 (62.7–73.5)	70.1 (63.9–73.4)	71.4 (66.5–75.7)	73.9 (69.1–77.0)	76.5 (70.0–79.4)	77.7 (71.2–80.9)	72.5 (66.9–73.7)

Values for individual participants are reported as mean (SD). For Wilcoxon signed-rank test, individuals with missing data for the weeks analyzed (participants 3, 6, 8, 10 and 12) had to be excluded from the statistical analysis.

∗ Individuals who developed gestational diabetes mellitus (GDM).

### Trends in physical activity and sleep across gestations

**FIGURE 6:**
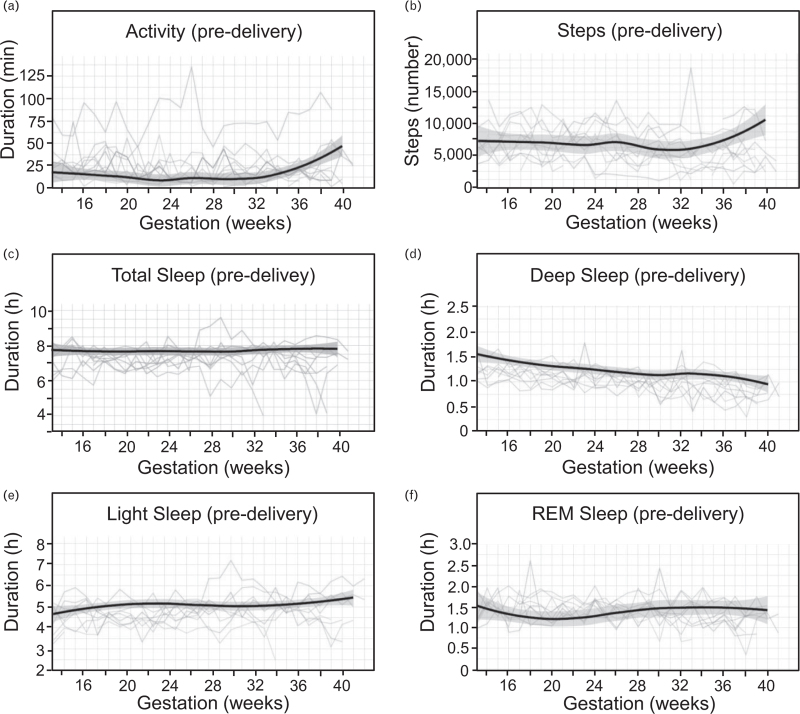
Sleep and activity patterns during pregnancy. Each colored line corresponds to an individual study participant. Color coding is conserved across the figure. Solid lines depict smoothed conditional means for daily: (a) activity (min); (b) number of steps taken; (c) total sleep duration (h); (d) duration of deep sleep (h); (e) duration of light sleep (h); (f) duration of REM sleep (h). Only predelivery values are included in the plots.

Continuous wearing of a smartwatch allowed us to generate individual physical activity and sleep pattern trajectories from week 13 to delivery. Activity was automatically detected and categorized by the smartwatch if the participant was burning three times as many calories as at rest. Our results indicated that while mean daily activity did not exceed 25 min until week 36, it did increase in the final weeks leading up to delivery (Fig. 6a). This was also reflected by an increase in the number of steps taken towards the end of pregnancy (Fig. 6b).

Analysis of sleep patterns (Fig. 6c–f) suggested that while there is little change in the Total Sleep duration across the pregnancy period (Fig. 6c), the amount of deep sleep decreased as pregnancy progressed (Fig. 6d).

## DISCUSSION

We report that remote monitoring of basic cardiovascular parameters in uncomplicated pregnancies is reliable and can generate readings that closely reflect those obtained during in-person clinical assessments. In this first multimodality study of recordings obtained from the comfort of own home, women took on average 4.6 measurements per modality each week, in contrast to the total of 11 measurements taken during the whole pregnancy according to current UK schedule of antenatal care for low-risk women [9]. Because of the high frequency of measurements taken, this allowed individualized trajectories to be generated that showed adaptation to various stages of pregnancy. In contrast to previous studies which elegantly demonstrated that home monitoring of blood pressure in pregnancy is feasible [36], our approach utilizes multimodality recording of various basic physiological parameters, which represents a novel approach. The high frequency of measurements covering the whole pregnancy allows not only to test established predictions on typical responses to pregnancy, but also depict individual's physiological adaptations at various pregnancy stages. Although different physiological trajectories were uncovered by this study, all study participants gave birth to healthy newborns. Thus, consistently with previous studies [36–38], home monitoring devices can be reliably used to monitor changes in cardiovascular physiology and body weight.

Our data indicates that when blood pressure is measured remotely, home recordings for both SBP and DBP dip at around 20 weeks of gestation. This is in agreement with the consensus in the field that blood pressure in healthy pregnancies gradually falls during first trimester [15,17,39], though recent studies suggest that in some individuals blood pressure remains largely static [40,41]. However, we show that while DBP returned back to early pregnancy values by week 30, SBP did not recover fully within this timeframe. Moreover, there was a pronounced but gradual increase in DBP from week 20 to week 35. These data also show that HR increases from early pregnancy to third trimester and subsequently drops in the postpartum period to values lower than at 13 weeks’ gestation. Given the limitations associated with the small sample size, it will be interesting for future larger studies to verify these observations.

Gestational hypertension affects 10% of pregnancies, may occur without warning and has wide ranging short and long term effects on maternal, fetal and infant health [42]. Moreover, gestation hypertension may be the first sign of the development of serious obstetric conditions, including preeclampsia [43]. We cannot comment on how early or reliably blood pressure changes consistent with preeclampsia would manifest themselves on daily monitoring, as none of the patients within our cohort consistently had blood pressure readings exceeding 140/90 mmHg or developed this condition. Considering that the incidence of preeclampsia is 2–8% [19], a larger cohort of women would be needed to examine these trends in more detail.

Frequent monitoring from home can also reveal patient specific trends, which deviate from cohort behavior. In particular, for 2/14 patients who completed the study, SBP remained persistently elevated during the 6 weeks of the postpartum period. Previous studies demonstrated that around 5.7% of preeclampsia presents de-novo in the postpartum period [44]. This pattern extends past the puerperium, and may have important consequences for postpregnancy maternal health [6].

Daily monitoring of maternal weight also revealed that almost 70% of the study cohort gained an amount of weight during pregnancy that guidelines [27] consider as “excessive”. Stratification of patients by prepregnancy BMI revealed that only 50% were within the recommended range: “normal” BMI = 18.5–24.9, as per World Health Organization guidelines [45]. Excessive weight gain during pregnancy often has life-long effects on body mass [46], and increases the risk of developing metabolic syndrome later in life [47]. In line with this, three of 14 study participants developed gestational diabetes. Interestingly, excessive weight gain was evident already in the first trimester. Considering that current antenatal care in the UK routinely offers appointments during the first trimester only at week 10, abnormal first trimester weight gain is often missed. In contrast, home-based monitoring offers a window of opportunity to detect such concerning results and act on them early. As evident from Smartwatch recordings, our study participants met the average daily activity level of 22 min that is recommended for pregnant women [48], hence the excessive gain weight was likely a result of a mismatched calorific intake.

Although the study provides a comprehensive picture of changes in maternal physiology from booking for antenatal care, its important limitation is that events during the earliest stages of pregnancy still remain unclear. As per the study protocol, recruitment of participants for this study occurred at the time of the conventional “booking visit”. Thus, events prior to 9–13 weeks of gestation have been missed. Another study demonstrated that individuals can be feasibly recruited to research studies preconception [49] and a similar approach may be employed in the future to better understand maternal cardiovascular physiology during early stages of the first trimester. Alternatively, self-monitoring devices could be offered to women instantly following a positive home pregnancy test. A comprehensive understanding of the first trimester is of particular importance, as this is likely when many of the critical changes occur. Our study suggests that studies can be performed from very early in pregnancy with high fidelity and that daily monitoring of maternal physiology could allow health in pregnancy to be stratified, with the intention of guiding individualized clinical decisions and personalized care. However, the relatively low adherence to the study protocol indicates that additional adjustments are necessary to make the frequent monitoring of multiple parameters compatible with the busy pregnancy and postpartum periods. In particular, fully automated data acquisition and charting could go a long way towards making multimodality recording in pregnancy feasible among a diverse cohort of pregnant women.

## ACKNOWLEDGEMENTS

Sources of funding: This research was supported by an NIHR Imperial Biomedical Research Centre (BRC) Clinical Lecturer grant to E.M. and was supported by Huma (Medopad) through supply of their app and physician portal. C.L. and E.M. are supported by National Institute for Health Research Comprehensive Biomedical Research Centre at Imperial College Healthcare NHS Trust and Imperial College London.

Disclaimers: None.

### Conflicts of interest

There are no conflicts of interest.

## Supplementary Material

Supplemental Digital Content

## Supplementary Material

Supplemental Digital Content
