# Supplementary material for: Remote multimodality monitoring of maternal physiology from the first trimester to postpartum period: study results
Source: J Hypertens. 2022 Aug 12;40(11):2280–91. doi: 10.1097/HJH.0000000000003260 (PMC9553246; doi:10.1097/HJH.0000000000003260)

**Supplementary Figure 1:** **Individual’s trends in HR, SBP and DBP across gestations captured by home recordings**

Examples of single patient’s home measurements for SBP (black), DBP (red), HR (blue) and clinic SBP/DBP recordings (green), obtained across gestations. Each plot summarizes an individual study participant.


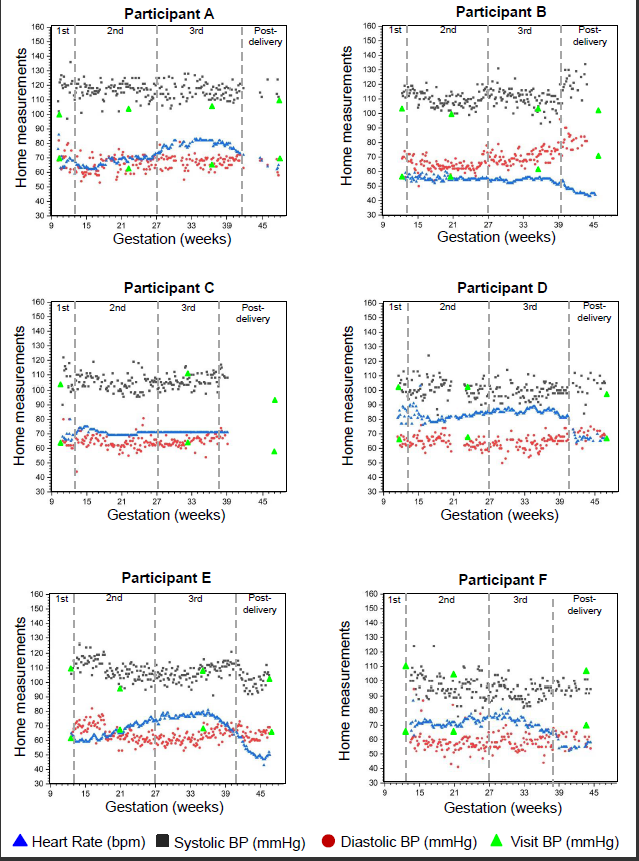

Supplement: Supplemental Digital Content [file jhype-40-2280-s001.doc]
